# COVID-19 Misinformation Detection: Machine-Learned Solutions to the Infodemic

**DOI:** 10.2196/38756

**Published:** 2022-08-25

**Authors:** Nikhil Kolluri, Yunong Liu, Dhiraj Murthy

**Affiliations:** 1 Computational Media Lab Department of Electrical and Computer Engineering The University of Texas at Austin Austin, TX United States; 2 School of Engineering College of Science and Engineering University of Edinburgh Edinburgh United Kingdom; 3 Computational Media Lab School of Journalism and Media, Moody College of Communication The University of Texas at Austin Austin, TX United States

**Keywords:** COVID-19, misinformation, machine learning, fact-checking, infodemiology, infodemic management, model performance, model accuracy, content analysis

## Abstract

**Background:**

The volume of COVID-19–related misinformation has long exceeded the resources available to fact checkers to effectively mitigate its ill effects. Automated and web-based approaches can provide effective deterrents to online misinformation. Machine learning–based methods have achieved robust performance on text classification tasks, including potentially low-quality-news credibility assessment. Despite the progress of initial, rapid interventions, the enormity of COVID-19–related misinformation continues to overwhelm fact checkers. Therefore, improvement in automated and machine-learned methods for an infodemic response is urgently needed.

**Objective:**

The aim of this study was to achieve improvement in automated and machine-learned methods for an infodemic response.

**Methods:**

We evaluated three strategies for training a machine-learning model to determine the highest model performance: (1) COVID-19–related fact-checked data only, (2) general fact-checked data only, and (3) combined COVID-19 and general fact-checked data. We created two COVID-19–related misinformation data sets from fact-checked “false” content combined with programmatically retrieved “true” content. The first set contained ~7000 entries from July to August 2020, and the second contained ~31,000 entries from January 2020 to June 2022. We crowdsourced 31,441 votes to human label the first data set.

**Results:**

The models achieved an accuracy of 96.55% and 94.56% on the first and second external validation data set, respectively. Our best-performing model was developed using COVID-19–specific content. We were able to successfully develop combined models that outperformed human votes of misinformation. Specifically, when we blended our model predictions with human votes, the highest accuracy we achieved on the first external validation data set was 99.1%. When we considered outputs where the machine-learning model agreed with human votes, we achieved accuracies up to 98.59% on the first validation data set. This outperformed human votes alone with an accuracy of only 73%.

**Conclusions:**

External validation accuracies of 96.55% and 94.56% are evidence that machine learning can produce superior results for the difficult task of classifying the veracity of COVID-19 content. Pretrained language models performed best when fine-tuned on a topic-specific data set, while other models achieved their best accuracy when fine-tuned on a combination of topic-specific and general-topic data sets. Crucially, our study found that blended models, trained/fine-tuned on general-topic content with crowdsourced data, improved our models’ accuracies up to 99.7%. The successful use of crowdsourced data can increase the accuracy of models in situations when expert-labeled data are scarce. The 98.59% accuracy on a “high-confidence” subsection comprised of machine-learned and human labels suggests that crowdsourced votes can optimize machine-learned labels to improve accuracy above human-only levels. These results support the utility of supervised machine learning to deter and combat future health-related disinformation.

## Introduction

### Background

Low information quality has led to adverse health outcomes for individuals during the COVID-19 pandemic [1-3]. Claims were being made on social media of dangerous home remedies and perceived preventative measures (eg, gargling with bleach-infused water) [4]. Low-quality and biased sources of information can be more alluring to some, as they easily capture attention and offer simpler solutions with unambiguous evidence. Due to their persuasive, “simple” messaging [2], these sources can appear more convincing to some because they confirm existing biases or better align with ideological narratives. Information veracity around COVID-19 is fundamentally important to the health outcomes of individuals worldwide [5]. For example, the information that has been circulated in social media spaces that masks do not prevent COVID-19 transmission or that wearing a mask is unhealthy [6] has been a major issue in terms of increased cases in the United States, but also in India, Brazil, and Turkey. Social media represent a key avenue where COVID-19–related disinformation and misinformation have been disseminated [7].

To tackle this misinformation, manual intervention alone is insufficient. Indeed, in the first quarter of 2020 alone, English-language fact checks of COVID-19–related content jumped 900% [8]. Despite checks increasing, there are a limited number of fact checkers. Moreover, they cannot check the high volume of content that needs evaluation [8]. Thus, creating any interventions for providing automated solutions to evaluate the credibility of COVID-19–related content being circulated remains critical.

In this study, we importantly compared COVID-19–related, general, and combined data sets for veracity classification applications, and developed a successful bidirectional long short-term memory (Bi-LSTM) machine-learning model (achieving internal and external validation accuracies of 93% and 75%, respectively). When crowdsourced human labels agreed with machine-learned outputs, the accuracy of 90% exceeded that of either approach alone. Our study provides critical, empirical evidence that small amounts of human labeling and machine learning can be an effective infodemic response to health disinformation.

### Misinformation and Disinformation

Misinformation is defined as “incorrect or misleading information” [9]. For example, a family member likely does not have intent to mislead you when they provide misinformation about politics or health, as they believe what they are sharing is actually true. Although misinformation is not inherently intentional, it can also cause real harm, as seen with COVID-19 misinformation being attributed to fatalities [10]. Disinformation refers to intentionally and surreptitiously disseminated false information aiming to obscure the truth [11]. Although both words refer to incorrect or inaccurate information, only disinformation is intentionally incorrect. A well-known example of a disinformation campaign is the 2016 Russian hacking of the Hillary Clinton campaign, and distribution of politically damaging propaganda on Facebook, Twitter, YouTube, and Instagram [12]. Russia’s social media disinformation campaign was found to have likely influenced the 2016 US election [13].

### COVID-19 and Social Media

Early COVID-19–related research was critical in documenting keywords, topics that were emerging, as well as temporal patterns [14-16]. Some work specifically highlighted instances of rumors [17], racism against individuals of Asian descent, and released data sets [18]. Other studies documented COVID-19–related misinformation and disinformation [8,19]. This work found that misinformation was widely diffused, which included that neem leaves can cure coronavirus [20], certain ethnic and racial groups were immune (particularly if they had darker skin), individuals in warmer countries would not be affected, and the disease was no more harmful than the common flu [21].

Other studies used machine-learned methods to try to classify misinformation and disinformation that was being circulated online [22-24]. By training machine-learned classifiers on labeled misinformation and disinformation data sets, these approaches were able to achieve accuracy ranging from 16.7% to 96% as measured by F1 scores. Early work was mostly focused on deploying rapid results rather than optimizing classifiers for the best accuracy to COVID-19–specific misinformation and disinformation. The presumption was that there would be a reasonable similarity of misinformation detection approaches more broadly with the misinformation being spread during COVID-19. As studies emerged, it became clear that COVID-19–specific data sets and platforms were needed.

### COVID-19–Related Misinformation Data Sets, Machine Learning, and Automated Detection

Due to the vast amount of COVID-19–related information circulating in public domains, automatic machine-learned identification and classification remains a critical method for detecting harmful content at scale. Six machine-learning algorithms with ensemble learning were used to study COVID-19–related Twitter data [25]. Combinations of several machine-learning approaches and natural language processing (NLP) are being used to develop large-scale misinformation detection. For example, ReCOVery, a repository for COVID-19 news credibility checking, evaluates various machine-learned methods [26]. One of the key issues hindering machine-learned methods remains the lack of large, verified, and labeled misinformation data sets [27]. A reason for this lack is that robust labeled data sets require involvement of humans with specific domain knowledge. Moreover, misinformation is a diverse dynamic phenomenon that changes rapidly [28]. Additionally, there remains a dearth of automated solutions that are scalable to incorporate content from multiple platforms. Although global studies indicate a high prevalence of misinformation (which disproportionately impacts low-income countries) [29], currently available data sets may not be large enough to be scalable [30].

To help address this gap, FakeCovid is a database of 5182 fact-checked news articles that uses 40 languages from 105 countries and classifies data using machine learning [31]. COVIDLIES is another database comprising 6761 expert-annotated COVID-19–related tweets [22]. Effective NLP methodology has also been used for detecting COVID-19 misinformation through YouTube videos by studying user comments [23]. More than 100 million Twitter messages have been collected and classified to build the “Infodemic Risk Index” to estimate the magnitude of exposure to misinformation across various regions and countries [2]. A manually labeled data set related to COVID-19 misinformation was released [32]. COVID-19–specific data sets have also been developed with non-English–language content, including Arabic [33], Portuguese [34], Italian [35], Chinese [36], and multiple Indic languages [37]. Machine-learned approaches have also been developed to complement manually labeled data sets related to COVID-19 [35].

### Machine-Learning Methods for Text Classification

NLP applications for text classification include news categorization, sentiment analysis, emotion detection, and authorship attribution [38,39]. Most classical machine-learning models in text classification tasks extract features (eg, bag of words) from the documents and then feed them to a classifier to make a prediction [38]. Note that, following prior works [40], we use the word “classical” to describe traditional supervised and unsupervised machine-learning methods.

The classical machine-learning models have some limitations, including tedious feature engineering in the process to extract hand-crafted features and the fact that they are difficult to generalize to new tasks due to their strong reliance on domain knowledge when designing features [38]. Deep-learning models make use of embedding models to map text into a feature vector with lower dimensions, thus limiting the need to rely on hand-crafted features (which often require domain knowledge) [38]. ELMo [41], a 3-layer Bi-LSTM model with 93 million parameters developed in 2017, achieved better performance than the previous most popular word2vec models [42,43] developed by Google in 2013. In 2018, OpenAI developed Generative Pre-trained Transformer (GPT) [42], and Google developed Bidirectional Encoder Representations from Transformers (BERT) [43], which inspired the creation of several different pretrained language models (PLMs) of large size based on transformers [38]. For example, XLNet, a generalized autoregressive pretraining method, allows for the learning of bidirectional contexts, and its autoregressive formulation overcomes some limitations of BERT [44]. Moreover, Facebook developed RoBERTa [45], which is trained on a larger data set than BERT. Large models based on transformers, including BERT, RoBERTa, and XLNet, achieved a high level of success in many NLP tasks [43-45].

### Objective

The objective of this study was to ameliorate the impact of online misinformation through automated, machine-learned, and scalable methods. Our study sought to answer the following three core research questions (RQs):

*RQ1*: Can approaches leveraging automated and scalable strategies such as machine learning, information retrieval, and crowdsourcing help combat misinformation when information growth exceeds fact-checker capabilities?

*RQ2*: Does training a machine-learning model on only COVID-19–related misinformation data, only on general misinformation data, or on both result in the highest performance on COVID-19–related data?

*RQ3*: Does combining crowdsourced labels with machine-learning model outputs improve accuracy over either approach individually?

## Methods

### Machine-Learned Classification

We first developed a classifier using the CoAID data set [46]; specifically, the 05-01-2020 and 07-01-2020 folders of the CoAID data set were used. Since there are more pieces of news deemed to be accurate (“true”) than those deemed to be inaccurate (“false”), we included all inaccurate news, but limited the quantity of true news to be equal to the amount of false news to have a balanced data set. For the Bi-LSTM model, we split our input data into a training set (75%) and test set (25%). Pandas [47] and scikit-learn [48] were used in our classifier development and implementation.

We evaluated different architectures, dropouts, activation functions, optimizers, regularizers, and batch sizes. We ultimately chose an embedding layer, Bi-LSTM layer, Dropout layer with a rate of 0.7, and Dense layer with a 1-dimensional output and sigmoid activation function. We used an Adam optimizer with a learning rate of 0.0001, binary cross-entropy loss, and a batch size of 1. The Bi-LSTM model has a kernel regularizer with *l*_1_ and *l*_2_ regularization factors of 1e-5 and 1e-4, respectively. In addition, we employed several state-of-the-art models for text classification, including PLMs such as BERT, RoBERTa, and XLNet. We selected RoBERTa, as it is an optimized BERT approach, and XLNet, as it is an autoregressive BERT-like model. We employed four transformers: BERT-base [43], XLNet [44], and two models fine-tuned on RoBERTa-base [45,49,50] for this specific classification task on the 7 data sets described in Table 1 for 3 epochs with default training arguments in HuggingFace Trainer [51]. Moreover, we trained a convolutional neural network (CNN) model for text classification [52], as this method has been extensively used in text classification [38].

All source code files for our models are publicly available as open source [53].

**Table 1:** Data set sources and specifications.

Data set	Source	Time range	Size (number of articles)				Type
			Noncredible news	True news	Total		
CoAID^a^	Tweets	Until May 1, 2020	572	1324	1896	COVID-19–specific	
FNN^b^	PolitiFact	N/A^c^	472	797	1270	General news	
FNN	Gossip Cop	N/A	16,818	5335	22,153	General news	
Validation data set 1^d^	Poynter.org (noncredible news); Washington Post, Associated Press, Politico (true news)	July 20, 2020, to August 8, 2020	3874	3177	7051	COVID-19–specific	
Validation data set 2^d^	Poynter.org (noncredible news); BBC, AXIOS, CBS News, The Globe and Mail (true news)	January 20, 2020, to June 15, 2022	14,398	16,232	30,630	COVID-19–specific	

^a^Only the 05-01-2020 folder of the CoAID data set was used.

^b^FNN: FakeNewsNet.

^c^N/A: not applicable.

^d^Scraped with the query term “COVID-19.”

### Data Evaluation

To develop our external validation data sets, we used data from Poynter [54], which had several thousand instances of COVID-19–related content with a “false” label. For “true” news, we inherited article accuracy from the credibility of the media source on which the documents were published, following an approach similar to the ReCOVery [26] and CoAID [46] COVID-19–related data sets. We created two external validation data sets with different “true” news sources to test the generalization ability of the models. The first external validation data set consists of ~4000 pieces of false-news content scraped from Poynter and ~3000 pieces of true-news content collected from several news outlets that we deemed to be reliable by inheriting source credibility. We used NewsAPI’s application programming interface [55] to retrieve content from the following news outlets: Reuters, BBC, The Wall Street Journal, The Washington Post, Associated Press, and Politico. We searched for articles from July 20, 2020, to August 8, 2020, with the query term “COVID-19.” With these parameters, we queried just over 3000 news articles and stored their labels, titles, sources, descriptions, URLs, and publication dates. The second external validation data set consists of ~14,000 pieces of noncredible news scraped from Poynter in the time range from March 20, 2020, to February 23, 2022, and ~16,000 pieces of true news scraped from BBC, AXIOS, CBS News, and The Globe and Mail with the query term “COVID-19” in the time range from January 20, 2020, to June 15, 2022. In total, after removing elements due to nonapplicable Poynter labels, the first data set had 7051 labeled pieces of COVID-19–related content within the time range from July 20, 2020, to August 8, 2020, and the second data set had 30,630 pieces of COVID-19–related content within the time range from January 20, 2020, to June 15, 2022.

We developed methods to evaluate whether training a machine-learning model on only COVID-19–related misinformation data, only on general misinformation data, or on both would result in the highest performance on new, unseen COVID-19 data sets. When evaluating general data sets, FakeNewsNet (FNN) [56,57] provided a data format matching our needs and with a sufficient volume for the scale of our training. For COVID-19–related data, we found that CoAID, a COVID-19 health care misinformation data set, with 1896 news articles, 183,564 related user engagements, 516 social platform posts about COVID-19, and ground truth labels [46], allowed us to achieve high internal validation accuracy in preliminary trials. To be as consistent across the two data sets as possible, we drew from standard benchmarking practices performed on data sets using default machine-learning model implementations. We trained on 7 different combinations of data sources to mimic different situations in the real world: (1) only CoAID, used to mimic the situation when sufficient topic-specific data are available; (2) partial (using only the 05-01-2020 folder of the CoAID data set) CoAID and FNN; (3) partial CoAID and PolitiFact; (4) partial CoAID and the GossipCop content from FNN, used to mimic the situation when we have a limited quantity of topic-specific data; (5) FNN; (6) PolitiFact; and (7) GossipCop, used to mimic the situation when no topic-specific data are available. For three classical models (support vector machine [SVM], logistic regression [LR], and Bernoulli naïve Bayes [BNB]) and six deep-learning models (Bi-LSTM, BERT-based model, two RoBERTa-based models [45,49,50], XLNet [44], and Text-CNN [52]) on all seven data source combinations, we computed precision, recall, and F1-score for both internal validation and the two external validation data sets described above. These were taken as a weighted average of both labels and rounded to the nearest hundredth, as detailed in Multimedia Appendix 1-3, and are available as a CSV file on our data repository [53].

### Ethics Considerations

The University of Texas at Austin Institutional Review Board (IRB) approved this study for human subjects research on April 20, 2021 (STUDY00000962). Informed consent from all study participants was obtained.

### Crowdsourced Classification

We recruited annotators from the crowdsourcing platform Prolific to vote on pieces of news content from the data set we created. On Prolific, we set the study distribution to “standard sample,” which launched the study to the whole participant pool [58]. In line with the IRB protocol, we limited voting to US residents only. We established approximately 10 rounds of Prolific tasks with each participant being paid varying amounts of ~$8 an hour, which resulted in 31,441 votes from 756 voters.

After completing the crowdsourced voting, we then processed the data both manually and with Python scripts for usability. We removed duplicate votes for the same label (two “true” votes) and votes from Prolific IDs that we could not find in the set of IDs reported to us by Prolific. The processed data set had more than 6800 pieces of content with at least 3 votes for either the “true” or “false” label. We took the initial ground truth labels from Poynter and credible news sources and mapped them to 0 or 1. “True” was coded as 1 and “false” was coded as 0. Additionally, “correct” labels were coded as 1 (2 labels), and all other labels were converted to 0 (690 labels). Mapping our labels to 0 or 1 allowed us to collect certain metrics for our data set. Some examples from the crowdsourced data set are provided in Table 2 (also see Multimedia Appendix 1). Voter soft labels of 0.0 or 1.0 indicate that the vote results are concordant (ie, all votes were for the same label), whereas a voter soft label range of 0.4-0.6 implies that (nearly) half of the voters have different opinions.

We also computed the percentage of agreeing decisions, which we defined as the probability that the label decided on by the crowdsourced votes was the same as the ground truth label. The percentage of agreeing decisions (human voter accuracy) was ~0.73, or 73%. We also calculated interannotator agreements to determine the agreement among voters. As the number of voters varied (from 3 to 7) for each piece of news content, Cohen and Fleiss κ statistics were not suitable for our data set. We therefore computed the percent agreement between users to determine interrater reliability (68.5%) for our data. As percent agreement does not take chance agreement into consideration, we calculated Krippendorff *α* (0.428). As percent agreement is considered to be acceptable when above 75% [59] and *α* is “acceptable at 0.667≤*α*≤0.823 and unacceptable at *α*<0.667” [60], there was low agreement among all voters in the crowdsourced data. Ultimately, crowdsourced voters had low accuracy (~73%) when identifying COVID-19–related noncredible content, and there was a high level of disagreement among them. Given that this data set was not used as the ground truth, but rather to evaluate whether labeled data from nonexperts could improve model performance, low agreement is not an issue for our use case. Moreover, this low agreement indicates that nonprofessionals respond to misinformation differently rather than consistently.

Given this high level of variability, we next evaluated whether our crowdsourced data could actually improve machine-learning model predictions. With this in mind, we developed and answered the following questions: (1) Which model best predicted crowdsourced labels? (2) Can model performance be improved after being blended with crowdsourced labels? (3) Which model performs best when blended with crowdsourced labels? (4) If we only take the subset of the data set where machine-learning models and human votes have agreeing labels, will the performance of prediction be improved?; if so, which model has the highest performance?

**Table 2:** Examples from the crowdsourced data set.

News title		Ground truth	Voter soft label^a^	Voter label	Total votes	Results
**Concordant human votes**						
	COVID-19 pandemic derails Germany’s push for migrant integration-Reuters	1	1.0	1	3	Correctly classified by humans
	Photo shows the last meeting of a Turkish doctor who died due to COVID-19 with his child in Munich	0	1.0	1	4	Misclassified by humans
	3M brings on another lobbying firm	1	1.0	1	5	Correctly classified by humans
	Video shows that the Italian government/Brisbane police used zombie robots/drones to chase their citizen and make them stay home	0	0.0	0	4	Correctly classified by humans
	British vaccine provokes immune response in first human studies	1	0.0	0	3	Misclassified by humans
	This video shows a woman eating a bat soup in Wuhan	0	0.0	0	5	Correctly classified by humans
**Discordant human votes**						
	An emergency department closed in a Spanish hospital	0	0.5	1	6	Misclassified by human
	Majority of Caledonian hotel jobs under review in Edinburgh	1	0.5	1	4	Correctly classified by humans
	England v Ireland: Captain Eoin Morgan relishes 'new journey' in ODI series	1	0.6	1	5	Correctly classified by humans
	Panic scene in Germany with people rushing into a supermarket	0	0.4	0	5	Correctly classified by humans

^a^Voter soft label is calculated by the number of true labels/total votes.

## Results

### Machine-Learned Classification

RQ1 asks whether automated systems can help combat COVID-19–related misinformation. We found that machine learning predicts veracity better than random. We developed a Bi-LSTM model trained on the CoAID data set. Specifically, we used 1257 entries from CoAID for training and tested our model on 419 entries from CoAID. We achieved a weighted average F1-score of 0.93 (with equal precision, recall, and accuracy) across both labels. Using the same model, the external validation results on our data set was an F1-score of 0.75, with equal precision, recall, and accuracy. In addition, we fine-tuned BERT-base, RoBERTa-fake-news, Fake-News-BERT-Detect, XLNet, and trained Text-CNN on 7 data set combinations and tested them on the two external validation data sets. The results are shown in Multimedia Appendix 1-2. We achieved accuracies of up to 91%, 93%, 97%, 94%, and 87% on the first external validation data set from BERT-base, RoBERTa-fake-news, Fake-News-BERT-Detect, XLNet, and trained Text-CNN, respectively. Accuracies of up to 93%, 84%, 93%, 91%, and 85% were achieved on the second external data sets from the same models. Given these results, RQ1 can be answered in the affirmative.

### Data Evaluation

RQ2 asks whether training a machine-learning model on only COVID-19–related misinformation data, on only general misinformation data, or on both results in the highest performance on COVID-19–related data. We found that machine-learned models benefit from COVID-19–related data. Specifically, after training on 7 different data sets (see Multimedia Appendix 1-3), RQ2 can be answered as follows: for classical models, the combination of topic-specific and general-topic data results in the best performance; however, pretrained models benefit from purely topic-specific data the most. In this study, we investigated the efficacy of three scenarios: (1) training on COVID-19–related misinformation, (2) training on non-COVID-19–related misinformation, and (3) training on both COVID-19–related misinformation and non-COVID-19–related misinformation. Our results indicate that including COVID-19–related misinformation (in our case CoAID data) helped—or, at least, maintained—model performance.

Examples of classical classification models include LR, SVM, BNB, hidden Markov model, and random forests [39]. In our experiment, classical models used included LR, SVM, and BNB. All three classical models shown in Multimedia Appendix 3 achieved the best accuracy when trained on the combination of CoAID and PolitiFact, whereas for deep-learning pretrained models, which have already “studied” the behavior of the English language, the best model performance was obtained when fine-tuned on CoAID only (see Multimedia Appendix 1-3). In instances where we are lacking additional COVID-19–related misinformation content, our findings suggest that incorporation of prior misinformation data sets in conjunction with COVID-19–specific misinformation data sets could potentially be useful to detect new COVID-19–related misinformation when using classical models. However, using PLMs (eg, BERT), which normally have much better performance on language tasks than classical models, fine-tuning on a topic-specific data set tended to give a better result. By combining COVID-19–related (ie, CoAID) and broad, multitopic misinformation data sets (ie, FNN, GossipCop, and PolitiFact), we evaluated the performance of our machine-learning models. Combining labeled data sets from different sources coupled with various machine-learning models is a novel contribution of our study in terms of producing a scalable and generalizable framework. As detailed in Multimedia Appendix 1-3, we found that the accuracy of models where we used only GossipCop data sets was very low. The lowest BNB accuracy we obtained (0.37) was also obtained for GossipCop, indicating the important role that labeled data sets play in the validity of misinformation detection. As GossipCop is considered a credible source of celebrity news, the labeled data sets of GossipCop are specific and have limited value to COVID-19 misinformation detection on their own. Conversely, combining CoAID and GossipCop as the input data to train our models significantly improved the accuracy (0.64) for the BNB model (Multimedia Appendix 3). As the best result, an accuracy of 96.55% was achieved when we fine-tuned Fake-News-BERT-Detect using only the CoAID data set (Multimedia Appendix 1). With these findings, RQ2 can be answered positively.

### Crowdsourced Classification

RQ3 asks whether combining crowdsourced labels with machine-learning model outputs improves accuracy over either approach individually. We found that combining human votes with machine-learned outputs allowed us to create higher performance models. Specifically, deep-learning models are able to predict human votes at an accuracy up to 70%. Combining human votes with machine-learned outputs allowed us to create a model with 99.1% accuracy. We achieved accuracy up to 98.59% when only considering the subset where model and human votes agreed.

We first evaluated how well our models could predict our crowdsourced values or the labels we generated from our Prolific labeling (see Multimedia Appendix 4-9). A label of 0 indicates that most voters voted false, while a label of 1 indicates that greater than or equal to half of the voters voted true. Using the models trained on the 7 data set combinations and testing on our data set of 7051 votes, the success at predicting the crowdsourced values from Prolific had accuracies up to 0.70 (see Multimedia Appendix 7). All values were rounded to the nearest hundredth.

Second, we blended the soft predictions (ie, probabilities) from the models and soft vote (combining the probabilities of each prediction in contrast to hard voting, which chooses the prediction that receives the most votes) results from crowdsourcing data in different proportions to assess both the maximum improvements and highest accuracies that can be achieved after blending. The soft vote results were computed by taking the number of votes for label 1 (credible) and dividing by the number of total votes. The results shown in Table 3 (predictions from blended models) were calculated by the following formula:

a×(soft predictions from model)+(1–a)×(soft vote results from crowdsourcing data)

Table 3 illustrates that models had higher accuracy on average after blending, and the highest accuracy we achieved was 99.1% on the first external validation data set (when blending 10% of user vote results with 90% of the machine-learning model prediction). Therefore, we found that models trained on general news were improved. Those models achieved much higher accuracies (up to 99.7%) after blending with user vote results. This represents a considerable improvement over the human vote accuracy of ~73%. As shown in Table 3, when a=0.9, the performance of Text-CNN trained on GossipCop could be improved from 42.6% to 99.1% after blending with crowdsourced data.

Third, as discussed in the Machine-Learned Classification section above, the machine-learning models had accuracies ranging from 41% to 98% and the human votes had approximately 73% accuracy. Out of the 7051 pieces of content, 39.24%-69.58% (for the best-performing model) showed agreement in both the human votes and the machine-learning model. We were therefore able to make reduced sets of 2766 to 4906 pieces of content. For each piece of content, we assigned its label to whichever value both the machine-learning model and human votes agreed on. Using this approach, our best accuracy was 98.59% (see Multimedia Appendix 10), which was from the Fake-News-BERT-Detect model fine-tuned on the CoAID data set. This is in comparison with an accuracy of 73% for human votes and 96.55% for the entire validation data set. All models achieved the best performance when the models were previously fine-tuned on COVID-19–specific data sets (ie, CoAID).

The performance of models trained/fine-tuned on a general-topic data set could be improved with crowdsourced data (eg, in low-data situations such as pandemics). Specifically, the base model achieved an accuracy of 71.01% on the whole validation data set. For example, for the subset, we achieved an accuracy of 89.96% at best (by BERT-base fine-tuned on PolitiFact). In addition, models trained on the combination general-topic and COVID-19–specific data set were also improved by this approach. Specifically, accuracies of up to 89.93% on the whole data sets (see Multimedia Appendix 1) were improved to up to 96.26% (for the subset). Practically speaking, both credibility tests could be applied to a piece of content and receive a label of “true” or “false” with up to 98.59% accuracy. Combining human votes with machine-learned outputs therefore outperformed models with human votes alone. Our response to RQ3 is that both blending crowdsourced labels with model predictions and reducing the data set to a “high-confidence” data subset increased model performance.

**Table 3:** Analysis of accuracy for blended models, evaluated on the first external validation data set.

Metric			a=0.9		a=0.7		a=0.5		a=0.3		a=0.1
	Average improvement	0.069		0.082		0.084		0.063		0.029	
**Maximum improvement**											
	Maximum improvement	0.565		0.562		0.463		0.385		0.415	
	Model name	Text-CNN trained on GossipCop		Text-CNN trained on GossipCop		Text-CNN trained on GossipCop		Text-CNN trained on GossipCop		Fake-News-BERT-Detect fine-tuned on GossipCop	
	Model accuracy (before blending)	0.426		0.426		0.426		0.426		0.302	
	Model accuracy (after blending)	0.991		0.981		0.889		0.804		0.717	
**Best performance**											
	Model name	Text-CNN trained on CoAID		Text-CNN trained on CoAID		Text-CNN trained on CoAID and PolitiFact		Text-CNN trained on GossipCop		Text-CNN trained on PolitiFact	
	Model accuracy (before blending)	0.874		0.874		0.798		0.426		0.499	
	Model accuracy (after blending)	0.991		0.984		0.891		0.804		0.728	

## Discussion

### Principal Results

Our results indicate that RQ1 (which asks whether automated systems and scalable strategies can help combat misinformation) can be answered in the affirmative. The models we trained showed an accuracy of 98% on our first external validation data set (of ~7000 posts and true news from July 20, 2020, to August 8, 2020) and an accuracy of 93% on our second validation data set (of ~15,000 posts and true news from January 20, 2020, to June 15, 2022). Labeling by fact-checkers can be time-consuming, labor-intensive, and expensive, whereas machine-learning models can be used at will and at scale once trained. These results support our finding that machine learning significantly improves fact checking given the reality that human fact-checkers are overburdened and cannot feasibly keep up with the increasing volume of online misinformation.

Regarding RQ2 (which asks what kind of data set is most helpful to machine learning), we found that training/fine-tuning on pandemic-specific content tends to result in higher accuracy. Specifically, our best-performing models were fine-tuned on COVID-19 topic content only. We evaluated three classical models and five deep-learning models trained on seven different data sets, including one topic-specific data set (CoAID only), three general-topic data sets (FNN, GossipCop, and PolitiFact), and three combinations of topic-specific and general-topic data sets (CoAID and FNN, GossipCop and CoAID, PolitiFact and CoAID). Classical models achieved the best accuracy when trained on a combination of general-topic and COVID-19–specific data (the combination of CoAID and PolitiFact), while deep-learning PLMs (eg, BERT), which have already been trained on English-language text and therefore could be considered as having “studied” the behavior of the English language, obtained the best model performance when fine-tuned on a COVID-19–specific data set (ie, CoAID).

Regarding RQ3, which asks whether combining crowdsourced labels with models can improve model performance, we found that blending crowdsourced labels with model predictions increased model performance. The blended model (crowdsourced votes mixed with a machine-learning model) was able to achieve an accuracy of 99.1%. Given that the accuracy of crowdsourced votes was 73% and the highest accuracy of our machine-learning models was 96.55%, our results therefore show that crowdsourcing can be used in conjunction with machine learning to boost accuracy. In addition, models trained on general news could be improved to achieve much higher accuracies after blending with user vote results. Specifically, we found improvements of up to 57.1% after blending (see Table 3). That being said, the performance of models trained/fine-tuned on a general-topic data set could only be improved when considering the subset. With neither crowdsourcing nor machine learning requiring time from expert fact-checkers, both are viable options for addressing COVID-19 and other health-related misinformation at scale.

### Future Work

Future work can further optimize our machine-learning model and extend and develop our labeled data set. Moreover, we hope that our findings encourage others to develop COVID-19–specific disinformation and misinformation data sets. As the quantity of COVID-19–related labeled data increases, the combination of COVID-19–related labeled data and general misinformation data should be further evaluated and benchmarked by others to enhance machine-learning model accuracy. Our results would therefore benefit from replication in future work with a data set consisting of both COVID-19–related and broad, multitopic content. Since we only crowdsourced votes for the first external validation data set (which spans one month), future work could crowdsource vote results on the second validation data set to strengthen the validity of our conclusions. Furthermore, the size of the crowdsourcing data set is relatively small (31,441 pieces of content and 4.46 average votes each), which could be strengthened with the accumulation of more votes and would increase the generalizability of our results. Thus, future work would benefit from extending our framework to a larger crowdsourced data set. Since collecting crowdsourced data could be time-consuming, using machine-learning models to generate pseudohuman votes can potentially be another way to strengthen the crowdsourced data set. After collecting crowdsourced data for a small news data set, the pseudohuman votes model trained on that data set can be used to predict human labels on a larger data set. This method would be especially useful with unlabeled news data sets, on which we could simulate human votes in the absence of ground truth labels.

Future work could also measure whether there are sufficient advantages of using machine-learning models rather than expert fact-checkers (given that the former method allows for cheaper and quicker large-scale data labeling). There is also the possibility that machine-learning models and professional fact-checkers combined together could deliver better results. For example, fact-checkers could use models to flag news to speed up their work, and the results from fact-checkers could be used to refine models. Human-in-loop models could be developed by using this method. A live news browser displaying news alongside fact-checker results or model predictions (if no fact-checker is available) could help assess credibility even when there is more misinformation than experts can check manually. Lastly, future work could further examine the relationship between crowdsourced outputs and ground truth labels for COVID-19–related data, a line of inquiry we minimally investigated in this study. Specifically, future work could examine when humans are more likely to make misjudgments by exploring the scenarios in which crowdsourced and ground truth labels are most likely to disagree. Research could explore crowdsourced data in different problem domains to identify the misinformation in problem domains that interventions should pay most attention to, using metrics such as the disagreement between human votes and ground truth labels.

### Limitations

A limitation of our work is that our study did not rigorously test the ceiling of possible model optimization on all combinations of FNN and CoAID models. Another minor limitation is that we assigned “false” to all labels (except two “correct” labels) in the Poynter data set when evaluating our model, even though a small portion of labels could be interpreted as true (<0.5% with labels such as “half true” and “mostly true”). The crowdsourced data set quality was potentially limited due to the number of votes per item and the time span of the labeled data set. Lastly, we were only able to crowdsource votes for the first external validation data set due to time and funding constraints.

### Conclusion

Manual fact checking is unable to cope with the large volumes of COVID-19–related misinformation that now exists [8]. To help address the proliferation of COVID-19–related misinformation, we developed an automated, machine-learned, and scalable approach. Since the best-performing models we evaluated were fine-tuned on COVID-19–specific content only, topic-specific data sets are much more helpful than general-topic data sets or the combination of the two. The 96.55% and 94.6% accuracy on the first and second external validation data set, respectively, suggest that machine learning can be used to achieve significantly better than random results for the difficult task of determining the veracity of COVID-19–related content. Our study also found that in the cases when only considering the reduced set of the content that both human votes and model outputs agreed on, the models achieved up to 99.1% accuracy. Models trained/fine-tuned on general-topic content can be improved to an acceptable level after combining with human votes, and may be used to supplement limited amounts of topic-specific content in low-data situations (eg, pandemics) to increase accuracy.

Our findings also suggest that machine-learning models can be augmented with the labels of lay, crowdsourced voters to boost accuracy without additional input from expert fact-checkers. Blending human votes with model prediction results achieved an accuracy up to 99.1% (by combining 10% of a human vote label with 90% of a label from the model). We have released our topic-related data set of 7000 ground truth and crowdsourced labels, machine-learning model, and code in open-source form to promote the development by others of automated, scalable solutions to the COVID-19 infodemic.

COVID-19 infodemic responses need to acknowledge that misinformation can be amorphous and highly decentralized. The machine-learned and automated approaches developed in this study rely on text features, making them powerful in that they can be extended (eg, by researchers or technology companies) to study a variety of platforms and contexts (eg, news and social media) in which online misinformation exists. Automation and machine learning offer the ability to exchange a small decrease in accuracy for scalability, which is an important consideration when misinformation growth exceeds fact-checking capabilities as continues to be the case during the COVID-19 pandemic.

## Appendix

Multimedia Appendix 1 Model performances on the first external validation data set.

Multimedia Appendix 2 Model performances on the second external validation data set.

Multimedia Appendix 3 Benchmarking results using classical models.

Multimedia Appendix 4 Results for the bidirectional long short-term memory (Bi-LSTM) model trained on CoAID and tested on crowdsourced labels.

Multimedia Appendix 5 Results for BERT-base tested on crowdsourced labels.

Multimedia Appendix 6 Results for RoBERTa-Fake-News tested on crowdsourced labels.

Multimedia Appendix 7 Results for Fake-News-BERT-Detect tested on crowdsourced labels.

Multimedia Appendix 8 Results for XLNet tested on crowdsourced labels.

Multimedia Appendix 9 Results for Text-CNN tested on crowdsourced labels.

Multimedia Appendix 10 Model performances on the reduced set of content when human and machine-learned votes agree.

## Abbreviations

BERT: Bidirectional Encoder Representations from Transformers
Bi-LSTM: bidirectional long short-term memory
BNB: Bernoulli naïve Bayes
CNN: convolutional neural network
FNN: FakeNewsNet
GPT: Generative Pre-trained Transformer
IRB: institutional review board
LR: logistic regression
NLP: natural language processing
PLM: pretrained language model
RQ: research question
SVM: support vector machine

## References

[1] Shim J, Lee Y, Ahn H (2021). A link2vec-based fake news detection model using web search results. Expert Syst Appl.

[2] Gallotti R, Valle F, Castaldo N, Sacco P, De Domenico M (2020). Assessing the risks of 'infodemics' in response to COVID-19 epidemics. Nat Hum Behav.

[3] Cinelli M, Quattrociocchi W, Galeazzi A, Valensise CM, Brugnoli E, Schmidt AL, Zola P, Zollo F, Scala A (2020). The COVID-19 social media infodemic. Sci Rep.

[4] Litman L, Rosen Z, Rosenzweig C, Weinberger-Litman SL, Moss AJ, Robinson J Did people really drink bleach to prevent COVID-19? A tale of problematic respondents and a guide for measuring rare events in survey data. MedRxiv.

[5] (2020). An ad hoc WHO technical consultation managing the COVID-19 infodemic: call for action, 7-8 April 2020. World Health Organization, Institutional Repository for Information Sharing.

[6] Khazan O (2020). How a bizarre claim about masks has lived on for months. The Atlantic.

[7] Bridgman A, Merkley E, Loewen P, Owen T, Ruths D, Teichmann L, Zhilin O (2020). The causes and consequences of COVID-19 misperceptions: understanding the role of news and social media. HKS Misinfo Review.

[8] Simon F, Howard PN, Nielsen RK (2020). Types, sources, and claims of COVID-19 misinformation. Reuters Institute.

[9] Misinformation. Merriam-Webster Dictionary.

[10] Coleman A (2020). 'Hundreds dead' because of Covid-19 misinformation. BBC.

[11] Disinformation. Merriam-Webster Dictionary.

[12] MacLellan K, Kerry F Britain says Russian troll factory is spreading disinformation on social media. Reuters.

[13] Jamieson KH (2020). Cyberwar: how Russian hackers and trolls helped elect a president: what we don't, can't, and do know.

[14] Banda JM, Tekumalla R, Wang G, Yu J, Liu T, Ding Y, Artemova E, Tutubalina E, Chowell G (2021). A large-scale COVID-19 Twitter chatter dataset for open scientific Research—an international collaboration. Epidemiologia.

[15] Chen E, Lerman K, Ferrara E (2020). Tracking social media discourse about the COVID-19 pandemic: development of a public coronavirus Twitter data set. JMIR Public Health Surveill.

[16] Gao Z, Yada S, Wakamiya S NAIST COVID: Multilingual COVID-19 Twitter and Weibo dataset. arXiv.

[17] Haouari F, Hasanain M, Suwaileh R ArCOV19-Rumors: Arabic COVID-19 Twitter dataset for misinformation detection. arXiv.

[18] He B, Ziems C, Soni S, Ramakrishan N, Yang D, Kumar S Racism is a virus: anti-Asian hate and counterspeech in social media during the COVID-19 crisis. arXiv.

[19] Kouzy R, Abi Jaoude J, Kraitem A, El Alam MB, Karam B, Adib E, Zarka J, Traboulsi C, Akl EW, Baddour K (2020). Coronavirus goes viral: quantifying the COVID-19 misinformation epidemic on Twitter. Cureus.

[20] (2020). FALSE: A claim that neem leaves can cure the novel coronavirus and relieve its symptoms has been shared thousands of times in multiple Facebook posts. Poynter.

[21] Singh L, Bansal S, Bode L, Budak C, Chi G, Kawintiranon K, Padden C, Vanarsdall R, Vraga E, Wang Y A first look at COVID-19 information and misinformation sharing on Twitter. ArXiv.

[22] Hossain T, Logan RI, Ugarte A (2020). COVIDLies: detecting COVID-19 misinformation on social media.

[23] Serrano JCM, Papakyriakopoulos O, Hegelich S (2020). NLP-based feature extraction for the detection of COVID-19 misinformation videos on YouTube.

[24] Dharawat A, Lourentzou I, Morales A, Zai CX Drink bleach or do what now? Covid-HeRA: A dataset for risk-informed health decision making in the presence of COVID19 misinformation. arXiv.

[25] Al-Rakhami MS, Al-Amri AM (2020). Lies kill, facts save: detecting COVID-19 misinformation in Twitter. IEEE Access.

[26] Zhou X, Mulay A, Ferrara E, Zafarani R ReCOVery: a multimodal repository for COVID-19 news credibility research. arXiv.

[27] Hua J, Shaw R (2020). Corona Virus (COVID-19) "infodemic" and emerging issues through a data lens: the case of China. Int J Environ Res Public Health.

[28] Zhang X, Ghorbani AA (2020). An overview of online fake news: characterization, detection, and discussion. Inf Process Manag.

[29] Cha M, Cha C, Singh K, Lima G, Ahn Y, Kulshrestha J, Varol O (2021). Prevalence of misinformation and factchecks on the COVID-19 pandemic in 35 countries: observational infodemiology study. JMIR Hum Factors.

[30] Asr F T, Taboada M (2019). Big Data and quality data for fake news and misinformation detection. Big Data & Society.

[31] Shahi GK, Nandini D FakeCovid--a multilingual cross-domain fact check news dataset for COVID-19. arXiv.

[32] Patwa P, Sharma S, Pykl S, Guptha V, Kumari G, Akhtar M, Ekbal A, Das A, Chakraborty T, Shu K, Bernard HR, Liu H, Akhtar MS (2021). Fighting an infodemic: COVID-19 fake news dataset. Combating Online Hostile Posts in Regional Languages during Emergency Situation. CONSTRAINT 2021. Communications in Computer and Information Science, vol 1402.

[33] Addawood A (2020). Coronavirus: Public Arabic Twitter Data Set. OpenReview.

[34] Melo Tde, Figueiredo CMS (2020). A first public dataset from Brazilian twitter and news on COVID-19 in Portuguese. Data in Brief.

[35] Rovetta A, Bhagavathula AS (2020). COVID-19-related web search behaviors and infodemic attitudes in Italy: infodemiological Study. JMIR Public Health Surveill.

[36] Yang C, Zhou X, Zafarani R (2021). CHECKED: Chinese COVID-19 fake news dataset. Soc Netw Anal Min.

[37] Kar D, Bhardwaj M, Samanta S, Azad AP No rumours please! A multi-indic-lingual approach for COVID fake-tweet detection. arXiv.

[38] Minaee S, Kalchbrenner N, Cambria E, Nikzad N, Chenaghlu M, Gao J (2022). Deep learning–based text classification. ACM Comput Surv.

[39] Alam T, Khan A, Alam F Bangla text classification using transformers. arXiv.

[40] Biamonte J, Wittek P, Pancotti N, Rebentrost P, Wiebe N, Lloyd S (2017). Quantum machine learning. Nature.

[41] Peters ME, Neumann M, Iyyer M, Gardner M, Clark C, Lee K, Zettle-Moyer L Deep contextualized word representations. arXiv.

[42] Radford A, Narasimhan K, Salimans T, Sutskever I (2018). Improving language understanding by generative pre-training. Amazon Simple Storage System (S3).

[43] Devlin J, Chang MW, Lee K, Toutanova K BERT: pre-training of deep bidirectional transformers for language understanding. arXiv.

[44] Yang Z, Dai Z, Yang Y, Carbonell J, Salakhutdinov R, Le QV (2019). XLNet: generalized autoregressive pretraining for language understanding.

[45] Liu Y, Ott M, Goyal N, Du J, Joshi M, Chen D, Levy O, Lewis M, Zettlemoyer L, Stoyanov V RoBERTa: a robustly optimized bert pretraining approach. arXiv.

[46] Cui L, Lee D CoAID: COVID-19 healthcare misinformation dataset. arXiv.

[47] The Pandas Development Team (2020). Zenodo.

[48] Abraham A, Pedregosa F, Eickenberg M, Gervais P, Mueller A, Kossaifi J, Gramfort A, Thirion B, Varoquaux G (2014). Machine learning for neuroimaging with scikit-learn. Front Neuroinform.

[49] Liu J (2022). Transformer-fake-news-detection. GitHub.

[50] Tatti GV (2021). roberta-fake-news. Hugging Face.

[51] Thomas W, Lysandre D, Victor S, Julien C, Clement D, Anthony M, Pierric C, Tim R, Remi L, Morgan F, Joe D, Sam S, Patrick VP, Clara M, Yacine J, Julien P, Canwen X, Teven LS, Sylvain G (2020). Transformers: State-of-the-Art Natural Language Processing.

[52] Gong M, Shou L, Lin W, Sang Z, Yan Q, Yang Z, Cheng F, Jiang D NeuronBlocks: building your NLP DNN models like playing Lego. arXiv.

[53] COVID-19 misinformation detection: machine-learned solutions to the infodemic. GitHub.

[54] (2020). Poynter.

[55] News API.

[56] Shu K, Mahudeswaran D, Wang S, Lee D, Liu H (2020). FakeNewsNet: A Data Repository with News Content, Social Context, and Spatiotemporal Information for Studying Fake News on Social Media. Big Data.

[57] Shu K, Sliva A, Wang S, Tang J, Liu H Fake news detection on social media: a data mining perspective. arXiv.

[58] Setting up a study on Prolific. Prolific.

[59] Inter-rater reliability IRR: definition, calculation. Statistics How To.

[60] Shabankhani B, Charati JY, Shabankhani K, Cherati SK (2020). Survey of agreement between raters for nominal data using Krippendorff's alpha. Arch Pharma Pract.

