# Supplementary material for: COVID-19 Misinformation Detection: Machine-Learned Solutions to the Infodemic
Source: JMIR Infodemiology. 2022 Aug 25;2(2):e38756. doi: 10.2196/38756 (PMC9987189; doi:10.2196/38756)
Supplement: Multimedia Appendix 1 [file infodemiology_v2i2e38756_app1.docx]

Multimedia Appendix 1. Model performances on the first external validation data set.

| Model / Accuracy | BERT-base | Roberta-fake-news | Fake-News-Bert-Detect | XLNet | Text-CNN |
| --- | --- | --- | --- | --- | --- |
| Out-of-box | 54.95% | 54.14% | 75.23% | 45.05% | N/A |
| CoAID | **91.40%** | **93.29%** | **96.55%** | **94.34%** | **87.39%** |
| FNN | 51.29% | 53.01% | 45.05% | 56.82% | 44.47% |
| CoAID & FNN | 81.83% | 80.67% | 88.75% | 82.38% | 72.13% |
| CoAID & PolitiFact | 79.21% | 89.93% | 88.61% | 82.48% | 79.84% |
| CoAID & GossipCop | 80.48% | 86.87% | 86.79% | 86.54% | 74.11% |
| GossipCop | 36.17% | 43.62% | 30.20% | 41.65% | 42.64% |
| PolitiFact | 71.01% | 69.16% | 75.77% | 70.16% | 49.93% |
