# Supplementary material for: COVID-19 Misinformation Detection: Machine-Learned Solutions to the Infodemic
Source: JMIR Infodemiology. 2022 Aug 25;2(2):e38756. doi: 10.2196/38756 (PMC9987189; doi:10.2196/38756)
Supplement: Multimedia Appendix 2 [file infodemiology_v2i2e38756_app2.docx]

Multimedia Appendix 2. Model performances on the second external validation data set.

| Model / Data source | BERT | Roberta-fake-news | Fake-News-Bert-Detect | XLNet | Text-CNN |
| --- | --- | --- | --- | --- | --- |
| Out-of-box | 47.01% | 39.27% | 48.79% | 47.06% | -% |
| CoAID | **94.56%** | **80.98%** | **93.05%** | **91.17%** | 85.36% |
| FNN | 53.55% | 46.91% | 52.99% | 66.51% | 56.36% |
| CoAID & FNN | 86.75% | 74.99% | 77.83% | 89.85% | 75.76% |
| CoAID & PolitiFact | 93.45% | 84.30% | 89.85% | 89.85% | **90.02%** |
| CoAID & GossipCop | 90.47% | 74.04% | 72.72% | 86.69% | 71.80% |
| GossipCop | 40.82% | 35.05% | 37.06% | 49.31% | 54.26% |
| Fine-tuned on PolitiFact | 84.10% | 74.53% | 75.95% | 79.42% | 70.43% |
