# Supplementary material for: COVID-19 Misinformation Detection: Machine-Learned Solutions to the Infodemic
Source: JMIR Infodemiology. 2022 Aug 25;2(2):e38756. doi: 10.2196/38756 (PMC9987189; doi:10.2196/38756)
Supplement: Multimedia Appendix 3 [file infodemiology_v2i2e38756_app3.docx]

Multimedia Appendix 3. Benchmarking results using classical models.

|  |  | Support Vector Machine | | | | Logistic Regression | | | | Bernoulli Naive Bayes | | | |
| --- | --- | --- | --- | --- | --- | --- | --- | --- | --- | --- | --- | --- | --- |
| Data Source | Validation | Prec | Rec | F1 | Acc | Prec | Rec | F1 | Acc | Prec | Rec | F1 | Acc |
| CoAID | Internal | 0.76 | 0.66 | 0.64 | 0.66 | 0.70 | 0.65 | 0.64 | 0.65 | 0.77 | 0.68 | 0.67 | 0.68 |
|  | External 1 | 0.67 | 0.62 | 0.61 | 0.62 | 0.68 | 0.62 | 0.61 | 0.62 | 0.71 | 0.64 | 0.63 | 0.64 |
|  | External 2 | 0.84 | 0.78 | 0.77 | 0.78 | 0.84 | 0.78 | 0.77 | 0.78 | 0.85 | 0.79 | 0.78 | 0.79 |
| CoAID + FNN | Internal | 0.53 | 0.53 | 0.53 | 0.53 | 0.52 | 0.52 | 0.52 | 0.52 | 0.53 | 0.52 | 0.50 | 0.52 |
|  | External 1 | 0.57 | 0.57 | 0.57 | 0.57 | 0.59 | 0.59 | 0.59 | 0.59 | 0.68 | 0.64 | 0.64 | 0.64 |
|  | External 2 | 0.65 | 0.65 | 0.65 | 0.65 | 0.69 | 0.69 | 0.69 | 0.69 | 0.85 | 0.81 | 0.81 | 0.81 |
| CoAID + PolitiFact | Internal | 0.68 | 0.68 | 0.67 | 0.68 | 0.66 | 0.65 | 0.65 | 0.65 | 0.69 | 0.68 | 0.67 | 0.68 |
|  | External 1 | 0.66 | 0.65 | 0.65 | **0.65** | 0.63 | 0.63 | 0.63 | **0.63** | 0.66 | 0.65 | 0.65 | **0.65** |
|  | External 2 | 0.85 | 0.83 | 0.83 | **0.83** | 0.84 | 0.83 | 0.82 | **0.83** | 0.86 | 0.83 | 0.83 | **0.83** |
| CoAID + GossipCop | Internal | 0.53 | 0.53 | 0.53 | 0.53 | 0.53 | 0.53 | 0.53 | 0.53 | 0.55 | 0.53 | 0.49 | 0.53 |
|  | External 1 | 0.55 | 0.55 | 0.55 | 0.55 | 0.55 | 0.55 | 0.55 | 0.55 | .65 | 0.65 | 0.65 | **0.65** |
|  | External 2 | 0.55 | 0.54 | 0.54 | 0.54 | 0.53 | 0.52 | 0.51 | 0.52 | 0.65 | 0.65 | 0.65 | 0.65 |
| FNN | Internal | 0.53 | 0.53 | 0.53 | 0.53 | 0.51 | 0.51 | 0.50 | 0.51 | 0.49 | 0.49 | 0.49 | 0.49 |
|  | External 1 | 0.53 | 0.51 | 0.50 | 0.51 | 0.57 | 0.54 | 0.53 | 0.54 | 0.64 | 0.64 | 0.64 | 0.64 |
|  | External 2 | 0.39 | 0.39 | 0.39 | 0.39 | 0.59 | 0.59 | 0.58 | 0.59 | 0.83 | 0.83 | 0.83 | **0.83** |
| PolitiFact | Internal | 0.72 | 0.72 | 0.72 | 0.72 | 0.71 | 0.71 | 0.71 | 0.71 | 0.72 | 0.72 | 0.72 | 0.72 |
|  | External 1 | 0.58 | 0.58 | 0.55 | 0.58 | 0.55 | 0.56 | 0.54 | 0.56 | 0.59 | 0.59 | 0.57 | 0.59 |
|  | External 2 | 0.83 | 0.82 | 0.82 | 0.82 | 0.79 | 0.79 | 0.79 | 0.79 | 0.80 | 0.79 | 0.79 | 0.79 |
| GossipCop | Internal | 0.51 | 0.50 | 0.50 | 0.50 | 0.52 | 0.52 | 0.51 | 0.52 | 0.53 | 0.53 | 0.53 | 0.53 |
|  | External 1 | 0.46 | 0.45 | 0.45 | 0.45 | 0.50 | 0.49 | 0.48 | 0.49 | 0.38 | 0.38 | 0.37 | 0.38 |
|  | External 2 | 0.53 | 0.53 | 0.53 | 0.53 | 0.37 | 0.37 | 0.37 | 0.37 | 0.18 | 0.18 | 0.18 | 0.18 |

*values in bold signify maximum external validation accuracy for each model
