# Supplementary material for: COVID-19 Misinformation Detection: Machine-Learned Solutions to the Infodemic
Source: JMIR Infodemiology. 2022 Aug 25;2(2):e38756. doi: 10.2196/38756 (PMC9987189; doi:10.2196/38756)
Supplement: Multimedia Appendix 4 [file infodemiology_v2i2e38756_app4.docx]

Multimedia Appendix 4. Results for the bidirectional long short-term memory (Bi-LSTM) model trained on CoAID and tested on crowdsourced labels.

|  | Precision | Recall | F1-score | Support |
| --- | --- | --- | --- | --- |
| 0 | 0.48 | 0.76 | 0.59 | 2757 |
| 1 | 0.76 | 0.48 | 0.58 | 4294 |
| Macro average | 0.62 | 0.62 | 0.59 | 7051 |
| Weighted average | 0.65 | 0.59 | 0.59 | 7051 |
| Accuracy | 0.59 | | | 7051 |
