# Supplementary material for: COVID-19 Misinformation Detection: Machine-Learned Solutions to the Infodemic
Source: JMIR Infodemiology. 2022 Aug 25;2(2):e38756. doi: 10.2196/38756 (PMC9987189; doi:10.2196/38756)
Supplement: Multimedia Appendix 5 [file infodemiology_v2i2e38756_app5.docx]

Multimedia Appendix 5. Results for BERT-base tested on crowdsourced labels.

|  | BERT-base | | | | | | | |
| --- | --- | --- | --- | --- | --- | --- | --- | --- |
| **Data source** | Out-of-box | CoAID | FNN | CoAID & FNN | CoAID & PolitiFact | CoAID & GossipCop | GossipCop | PolitiFact |
| **Accuracy** | 0.39 | **0.66** | 0.51 | 0.60 | 0.58 | 0.58 | 0.47 | 0.53 |
| **F1 Score** | 0.00 | 0.65 | 0.57 | 0.55 | 0.51 | 0.52 | 0.56 | 0.43 |
| **Precision** | 0.00 | 0.86 | 0.61 | 0.86 | 0.88 | 0.86 | 0.56 | 0.82 |
| **Recall** | 0.00 | 0.53 | 0.53 | 0.41 | 0.36 | 0.37 | 0.56 | 0.29 |
