# Supplementary material for: COVID-19 Misinformation Detection: Machine-Learned Solutions to the Infodemic
Source: JMIR Infodemiology. 2022 Aug 25;2(2):e38756. doi: 10.2196/38756 (PMC9987189; doi:10.2196/38756)
Supplement: Multimedia Appendix 6 [file infodemiology_v2i2e38756_app6.docx]

Multimedia Appendix 6. Results for RoBERTa-Fake-News tested on crowdsourced labels.

|  | Roberta-fake-news | | | | | | | |
| --- | --- | --- | --- | --- | --- | --- | --- | --- |
| **Data source** | Out-of-box | CoAID | FNN | CoAID & FNN | CoAID & PolitiFact | CoAID & GossipCop | GossipCop | PolitiFact |
| **Accuracy** | 0.63 | **0.68** | 0.58 | 0.59 | 0.66 | 0.64 | 0.60 | 0.60 |
| **F1 score** | 0.75 | 0.68 | 0.67 | 0.54 | 0.65 | 0.62 | 0.75 | 0.64 |
| **Precision** | 0.64 | 0.87 | 0.64 | 0.84 | 0.87 | 0.87 | 0.61 | 0.70 |
| **Recall** | 0.90 | 0.56 | 0.69 | 0.40 | 0.52 | 0.48 | 0.98 | 0.59 |
