# Supplementary material for: COVID-19 Misinformation Detection: Machine-Learned Solutions to the Infodemic
Source: JMIR Infodemiology. 2022 Aug 25;2(2):e38756. doi: 10.2196/38756 (PMC9987189; doi:10.2196/38756)
Supplement: Multimedia Appendix 7 [file infodemiology_v2i2e38756_app7.docx]

Multimedia Appendix 7. Results for Fake-News-BERT-Detect tested on crowdsourced labels.

|  | Fake-News-Bert-Detect | | | | | | | |
| --- | --- | --- | --- | --- | --- | --- | --- | --- |
| **Data source** | Out-of-box | CoAID | FNN | CoAID & FNN | CoAID & PolitiFact | CoAID & GossipCop | GossipCop | PolitiFact |
| **Accuracy** | 0.57 | **0.70** | 0.61 | 0.65 | 0.65 | 0.63 | 0.47 | 0.60 |
| **F1 score** | 0.49 | 0.71 | 0.76 | 0.64 | 0.64 | 0.62 | 0.61 | 0.58 |
| **Precision** | 0.87 | 0.86 | 0.61 | 0.86 | 0.87 | 0.85 | 0.55 | 0.78 |
| **Recall** | 0.34 | 0.60 | 1.00 | 0.51 | 0.50 | 0.48 | 0.69 | 0.47 |
