# Supplementary material for: COVID-19 Misinformation Detection: Machine-Learned Solutions to the Infodemic
Source: JMIR Infodemiology. 2022 Aug 25;2(2):e38756. doi: 10.2196/38756 (PMC9987189; doi:10.2196/38756)
Supplement: Multimedia Appendix 9 [file infodemiology_v2i2e38756_app9.docx]

Multimedia Appendix 9. Results for Text-CNN tested on crowdsourced labels.

|  | Text_CNN | | | | | | |
| --- | --- | --- | --- | --- | --- | --- | --- |
| **Data Source** | CoAID | FNN | CoAID & FNN | CoAID & PolitiFact | CoAID & GossipCop | GossipCop | PolitiFact |
| **Accuracy** | **0.64** | 0.51 | 0.61 | 0.59 | 0.60 | 0.56 | 0.47 |
| **F1 Score** | 0.64 | 0.61 | 0.65 | 0.55 | 0.63 | 0.70 | 0.46 |
| **Precision** | 0.83 | 0.59 | 0.72 | 0.82 | 0.73 | 0.60 | 0.60 |
| **Recall** | 0.52 | 0.63 | 0.59 | 0.41 | 0.55 | 0.85 | 0.37 |
