# Supplementary material for: COVID-19 Misinformation Detection: Machine-Learned Solutions to the Infodemic
Source: JMIR Infodemiology. 2022 Aug 25;2(2):e38756. doi: 10.2196/38756 (PMC9987189; doi:10.2196/38756)
Supplement: Multimedia Appendix 10 [file infodemiology_v2i2e38756_app10.docx]

Multimedia Appendix 10. Model performances on the reduced set of content when human and machine-learned votes agree.

| **Model Name** | **BERT-base** | | | | | | | |
| --- | --- | --- | --- | --- | --- | --- | --- | --- |
| **Data source** | Out-of-box | CoAID | FNN | CoAID & FNN | CoAID & PolitiFact | CoAID & GossipCop | GossipCop | PolitiFact |
| **Accuracy** | 84.15% | **97.31%** | 72.64% | 94.31% | 93.68% | 94.49% | 58.77% | 89.96% |
| **Label agreed** | 39.24% | 66.09% | 50.68% | 59.90% | 57.99% | 58.14% | 46.58% | 53.17% |
| **Model Name** | **roberta-fake-news** | | | | | | | |
| **Data source** | Out-of-box | CoAID | FNN | CoAID & FNN | CoAID & PolitiFact | CoAID & GossipCop | GossipCop | PolitiFact |
| **Accuracy** | 70.58% | **97.27%** | 71.24% | 93.98% | 96.26% | 95.25% | 62.73% | 83.73% |
| **Label agreed** | 62.74% | 68.12% | 57.95% | 58.92% | 65.97% | 64.11% | 60.04% | 60.14% |
| **Model name** | **Fake-News-Bert-Detect** | | | | | | | |
| **Data source** | Out-of-box | CoAID | FNN | CoAID & FNN | CoAID & PolitiFact | CoAID & GossipCop | GossipCop | PolitiFact |
| **Accuracy** | 91.08% | **98.59%** | 63.74% | 95.89% | 95.91% | 95.61% | 52.28% | 89.64% |
| **Label agreed** | 56.94% | 69.58% | 60.76% | 65.27% | 65.24% | 63.49% | 46.90% | 59.62% |
| **Model name** | **XLNet** | | | | | | | |
| **Data source** | Out-of-box | CoAID | FNN | CoAID & FNN | CoAID & PolitiFact | CoAID & GossipCop | GossipCop | PolitiFact |
| **Accuracy** | 63.74% | **97.97%** | 73.56% | 94.80% | 94.90% | 95.19% | 64.16% | 85.90% |
| **Label agreed** | 60.76% | 68.21% | 60.26% | 59.96% | 60.00% | 63.87% | 47.59% | 57.72% |
| **Model name** | **Text_CNN** | | | | | | | **BiLSTM** |
| **Data source** | CoAID | FNN | CoAID & FNN | CoAID & PolitiFact | CoAID & GossipCop | GossipCop | PolitiFact | CoAID |
| **Accuracy** | **95.62%** | 66.13% | 85.72% | 93.62% | 87.74% | 62.83% | 73.31% | 90.01% |
| **Label agreed** | 64.27% | 50.69% | 61.13% | 58.79% | 60.25% | 56.45% | 46.87% | 58.74% |
